# Antibody Response Following a Two-Dose mRNA Vaccination Regimen, in Health Care Workers of a Tertiary Hospital in Athens, Greece

**DOI:** 10.3390/jpm11060576

**Published:** 2021-06-19

**Authors:** Elissavet Kontou, Kyriaki Ranellou, Dimitrios Zoulas, Anastasia Bletsa, Eirini Rompola, Evangelia-Theophano Piperaki, Nikolaos Athanasiou, Kleio Ampelakiotou, Maria Pratikaki, Christina Stergiopoulou, Athina Argyropoulou, Androula Alevra, Aggeliki Megalou, Alexandra Tsirogianni

**Affiliations:** 1 Immunology-Histocompatibility Department, “Evangelismos” General Hospital, 10676 Athens, Greece; kontolisa@gmail.com (E.K.); anable_3@yahoo.com (A.B.); k.ampelakiotou@gmail.com (K.A.); alextsir@gmail.com (A.T.); 2 Microbiology Department, “Evangelismos” General Hospital, 10676 Athens, Greece; athina.argyropoulou@gmail.com; 3 Blood Bank Department, “Evangelismos” General Hospital, 10676 Athens, Greece; dzoulas@gmail.com (D.Z.); cstergio73@yahoo.gr (C.S.); megalouageliki8@gmail.com (A.M.); 4 Clinical Biochemistry Department, “Evangelismos” General Hospital, 10676 Athens, Greece; erobola@hotmail.com (E.R.); mpratikaki@yahoo.com (M.P.); aaprokopiou@hotmail.com (A.A.); 5 Department of Microbiology, Medical School, National and Kapodistrian University of Athens, 11527 Athens, Greece; epiper@med.uoa.gr; 6 St ICU, “Evangelismos” General Hospital, 10676 Athens, Greece; nikolaosathanasiou14@gmail.com

**Keywords:** SARS CoV-2, mRNA vaccine, antibody response, healthcare workers

## Abstract

We analyzed the antibody responses of 564 hospital workers in Athens, Greece, after vaccination with two doses of the BNT162b2 (Comirnaty^®^; BioNTech and Pfizer) mRNA COVID-19 vaccine. A greater antibody increase was observed in women, younger age groups, previously infected individuals and personnel working in COVID-19 clinics. Notably, individuals with a prior COVID-19 infection mounted a significantly higher antibody titer following the first dose than the rest of the population; the same was true for those working in COVID-19 clinics, even without history of previous infection.

## 1. Introduction

Vaccines against the SARS-CoV-2 virus show great promise in limiting/controlling the spread of the virus and subsequently curbing the pandemic. Data on immune responses to the disease and the vaccine in populations worldwide are becoming increasingly available [1,2]. Emerging serological data following vaccination will enhance our understanding of the immune response to the vaccine and influence vaccination strategies, depending on population characteristics, including prior infection with the virus. In the present study, we present results from an ongoing large-scale assessment of antibody responses following a complete, two-dose vaccination regimen with the BNT162b2 (Comirnaty^®^; BioNTech and Pfizer) mRNA vaccine. To the best of our knowledge, this is the first report of a large-scale antibody assessment in Greek healthcare professionals following vaccination.

## 2. Materials and Methods

The population examined consisted of 564 healthcare workers. Enrollment was open to all hospital personnel scheduled for vaccination and not restricted by any pre-specified criteria. Of those, 170 (30.2%) were male and 394 (69.8%) female, 272 (48.2%) were doctors, 134 (23.8) were nurses, 45 (8%) were administrative staff, 64 (11.3%) were technicians and 49 (8.7%) other (pharmacists, biologists, dentists, etc.) (Table 1).

Thirteen individuals (2.3%), 9 male and 4 female, which included 1 dentist, 8 doctors, 3 nurses and 1 member of administrative staff, had a prior confirmed COVID-19 infection in the 3–11 months prior to vaccination. A proportion (94/564 16.7%) of the doctor and nurse population tested was employed in wards devoted exclusively to the care of COVID-19 patients (Intensive Care Unit (ICU), Critical Care Unit (CCU), COVID-19 clinics). All individuals received two doses of the mRNA Pfizer-BioNTech vaccine. Data on prior COVID-19 infection and symptoms experienced after each dose were collected for all participants. Antibody concentrations were assessed at two time points: 21 ± 1 days after the first dose and 24 ± 2 days after the second dose. Levels of circulating SARS CoV-2 anti-spike IgG(S) and anti-nucleocapsid IgG(N) antibodies were quantified using the Abbott Diagnostics SARS-CoV-2 IgG chemiluminescent microparticle immunoassay (Abbott Diagnostics, Abbott Park, Illinois) on an Abbott Diagnostics Architect i2000 SR and an Alinity i analyzer, according to the manufacturer’s instructions. Results were expressed in AU/mL and were interpreted as positive if ≥50 [3]. Informed consent was obtained from all participants and the study was approved by the Institutional Review Board of “Evangelismos” General Hospital (PN 9/21-01-21).

### Statistical Analysis

Normally distributed variables were expressed as means (standard deviation). Antibodies for COVID-19 were expressed as geometric means titers (GMT) with 95% confidence intervals. Qualitative variables were expressed as absolute and relative frequencies. Repeated-measures analysis of variance (ANOVA) was adopted to evaluate the changes observed in antibody titers over the follow-up period and to associate them with their characteristics. Bonferroni correction was used in the case of multiple testing in order to control for type I error. Logarithmic transformations were used for repeated-measures analysis of variance due to non-normal distribution. Spearman correlation coefficients were used to explore the association of pre- and post-second dose antibody levels. All reported *p*-values are two-tailed. Statistical significance was set at *p* < 0.05 and analyses were conducted using Stata statistical software, version 13.0 ((Stata Cooperation, College Station, TX, USA))

## 3. Results

**Figure 1:**
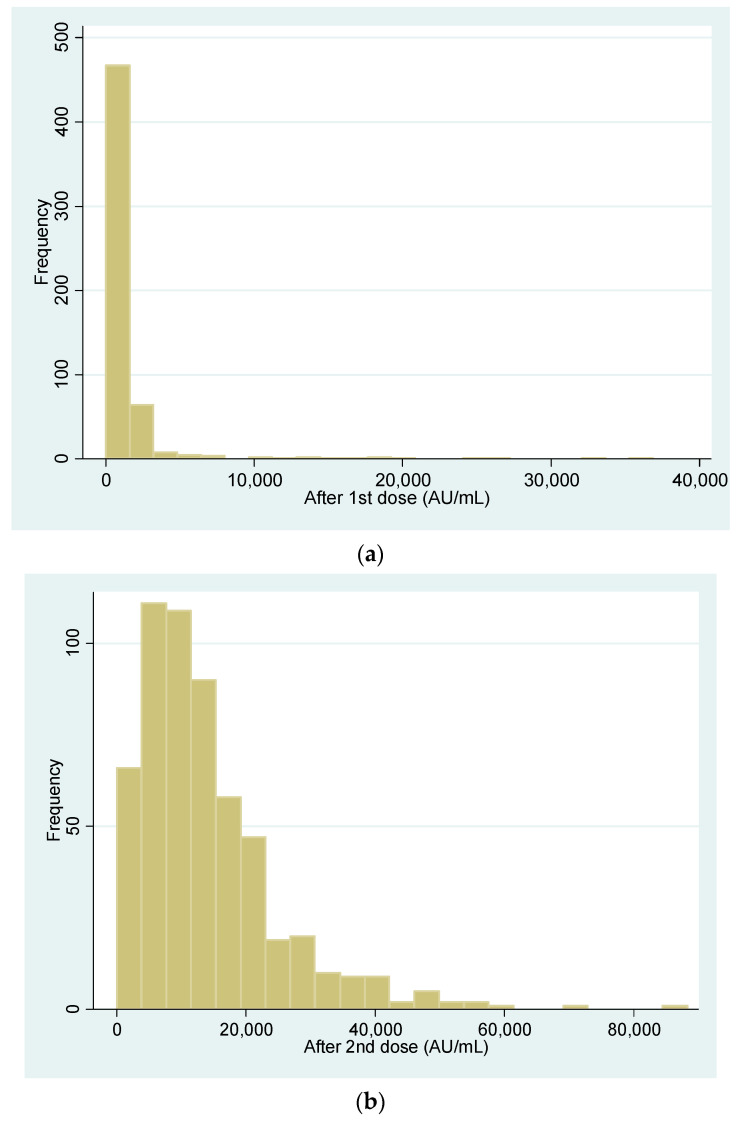

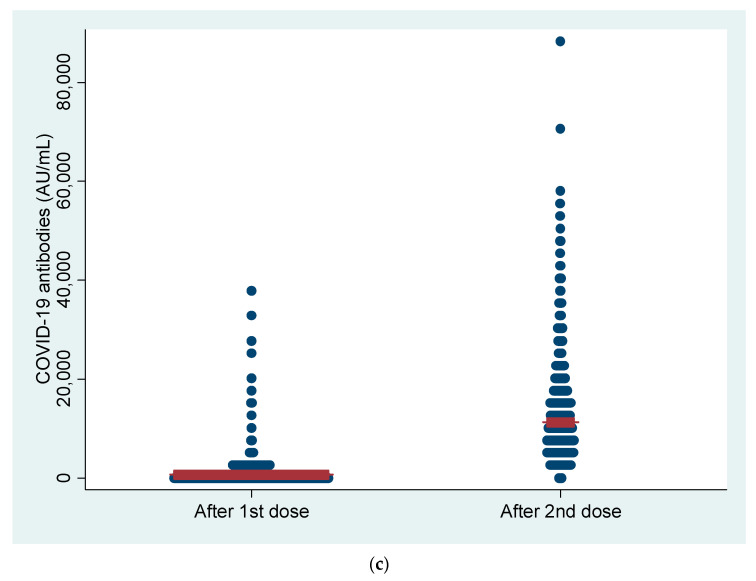
(**a**) Antibody concentration after the first dose. (**b**) Antibody concentration after the second dose. (**c**) Dot plot for antibody titers before and one month after the second dose. Note: red line represents the GMT (Geometric Means Titers).

Vaccine recipients (*n* = 564) included 170 (30.2%) male and 394 (69.8%) female individuals aged 48.6 ± 10.1 years. After the first dose, 2/564 individuals (0.4%) did not have a detectable antibody response. One of the two, a kidney transplant recipient, did not mount an antibody response even after the second dose, while the other, a patient with common, variable immune deficiency (CVID), responded, bringing the final percentage of participants with an antibody response to a total of 99.8% (563/564). Descriptive statistics for antibody responses following the first and second vaccine dose are shown in Table 2 and in Figure 1a,b. The antibody GMTs after the first and the second dose were 583.3 and 10,294.5, respectively, showing a significant antibody titer increase between the first and second dose in all groups (*p* < 0.001) (Table 2, Figure 1c).

Changes in antibody levels during follow-up for different groups are shown in Table 3. The antibody titer increase was less than 10-fold in 20.9%, 10- to 30-fold in 57% and more than 30-fold in 22.1% of the participants. A significant correlation was found between titers after the first and second dose measurements (Spearman’s *r* = 0.68, *p* < 0.001).

At both time points, women, younger age groups, participants with a previous COVID-19 infection and those working in COVID-19 clinics had significantly higher antibody levels. After the second dose, antibody titers significantly increased in all study groups. As indicated by the interaction effect of the analysis (*p3* values, Table 3), the degree of increase was significantly greater in women compared to men, in younger participants and in those who worked in COVID-19 clinics, but not in previously infected individuals. This group, however, had consistently higher antibody titers than those who had not been previously infected across all measurements. Moreover, at both time points, individuals that experienced fever or any adverse event after vaccination had higher antibody levels.

Post-vaccine adverse effects were also assessed, in parallel with antibody response analysis. In total, 342/564 subjects (67.2%) experienced at least one symptom after administration of the second dose, including fatigue (172/564, 33.9%), muscle pain (136/564, 26.7%), erythema at the injection site (133/564, 26.1%), headache (123/564, 24.2%), fever (87/564, 17.1%), lymphadenitis (24/564, 4.7%), allergic reactions (4/564, 0.8%) and severe allergic reaction with laryngeal edema (1/564, 0.18%). Adverse events were not found to be significantly different in previously infected participants compared to those not previously infected (*p* > 0.05).

## 4. Discussion

In the present study, we measured IgG(S) in healthcare professionals working in a large tertiary care hospital in Athens, Greece at two time points, following the first and second dose of the BNT162b2 (Pfizer–BioNTech) mRNA vaccine. To the best of our knowledge, this is the first large-scale assessment of antibody responses in vaccinated healthcare professionals in Greece. The most noteworthy findings of our study are the following. Overall, only one participant (1/564, 0.18%) failed to mount an antibody response following both doses of the vaccine and was therefore considered to be a non-responder. The high rate of vaccine antibody response (99.82%) could possibly be attributed to the young age and health status of the examined population. As expected, vaccinated healthcare professionals showed a significant rise in antibody titers between the first and the second dose [4], with a greater antibody increase observed in women, younger individuals, previously infected individuals and personnel working in COVID-19 clinics. A significant correlation was found between titers after the first and second dose measurements (*p* < 0.001). Thus, individuals with a higher antibody response after the first dose exhibited a similarly higher response following the second, which might be attributed to host-specific immune responses.

Notably, previously infected individuals displayed significantly higher antibody titers following the first vaccine dose, in comparison to the rest of the study population, which has also been reported previously [5,6]. This has been attributed to a booster effect of the vaccine on preexisting natural immunity in these individuals [4]. The degree of increase was significantly greater in women compared to men, in younger individuals and in those that worked in COVID-19 clinics, but not in previously infected individuals, possibly due to this group’s small size (*n* = 13).

Surprisingly, a significantly higher antibody titer following the first vaccine dose was also observed in the COVID-19 clinic group. We attempted to establish whether these individuals had been previously infected (possibly asymptomatically and therefore unknowingly) by measuring IgG(N) levels, but all results were negative. It should, however, be borne in mind that a lack of detectable IgG does not exclude previous infection, as their concentrations are known to decline over time [7,8]. Thus far, this finding, although quite intriguing, cannot be fully explained. Additional studies that include the baseline serological status of prospective vaccinees, including those in continuous previous close contact with COVID-19 patients, might help to elucidate this observation.

Limitations of our study include the following. Baseline antibody titers were not measured for any of the participants, nor were neutralizing antibodies and cell-mediated immunity assessed. We focused on BNT162b2 vaccinees and it is possible that responses to other vaccines may vary. Moreover, data collected on medical history, exposure and adverse effects were all self-reported and not available for a significant proportion of the previously infected group of vaccinees. Finally, adverse effects were recorded by participants only following the second vaccine dose.

As immunization of the population expands, continuing assessment of antibody levels will enhance our understanding of immune responses to the virus and might help to improve national vaccination strategies in the future. The results of our ongoing study, with the inclusion of additional healthcare workers and follow-up antibody assessment for all participants, will be available shortly.

## Figures and Tables

**Table 1:** Sample characteristics.

Sex		*n*	(%)
	Men	170	30.1
	Women	394	69.9
Age, mean (SD ^a^)		48.6	10.1
Age			
	<42	125	24.5
	42–48	126	24.7
	49–55	124	24.3
	>55	135	26.5
Occupation			
Doctor		272	48.2
	Nurse	134	23.8
	Administrative personnel	45	8.0
	Technician	64	11.3
	Other	49	8.7
Prior COVID-19 infection		13	2.3

^a^ SD: Standard Deviation.

**Table 2:** Descriptive statistics for antibodies after the first and the second dose.

	Minimum	Maximum	Median (IQR)	GMT (95% CI)
After 1st dose	0.0	36,878.0	635 (289.5–1225.8)	583.3 (524.6–648.6)
After 2nd dose	0.0	88,292.0	11,262.5 (6352.1–18,149.9)	10,294.5 (9558.4–11,087.2)

IQR: Interquartile Range, GMT: Geometric Means Titers, CI: Confidence Interval.

**Table 3:** Changes in antibody levels during follow-up.

		After 1st Dose	After 2nd Dose	Change	*p* ^2^	*p* ^3^
		GMT (95% CI)	GMT (95% CI)	GMT		
Sex	Women	658.0 (582.8–743.0)	10,906.4 (9953.9–11,950.2)	10,248.4	<0.001	0.022
	Men	441.8 (359.1–543.4)	9011.0 (7956.3–10,205.4)	8569.2	<0.001	
	*p* ^1^	0.001	0.020			
Age	<42 ^a^	964.9 (811.8–1146.9) ^c.d^	13,800.5 (12,341.7–15,431.7) ^c.d^	12,835.6	<0.001	<0.001
	42–48 ^b^	664.8 (543.3–813.5) ^d^	10,544.6 (9202.6–12,082.4) ^d^	9879.8	<0.001	
	49–55 ^c^	520.9 (418.6–648) ^a.d^	9348.9 (7619.8–11,470.4) ^a^	8828.0	<0.001	
	>55 ^d^	340.7 (262.7–442) ^a.b.c^	7867.6 (6756.2–9161.8) ^a.b^	7526.9	<0.001	
	*p* ^1^	<0.001	<0.001			
Occupation	Doctor	590.3 (496.7–701.4)	10,142.9 (8992–11,441.2)	9552.6	<0.001	0.277
	Nurse	634.1 (531.1–757)	10,745.3 (9435–12,237.5)	10,111.2	<0.001	
	Administrative	421.5 (275.1–645.7)	9851.8 (7668.3–12,657.2)	9430.3	<0.001	
	Technicians	624.5 (490.2–795.6)	10,347.9 (8571.3–12,492.6)	9723.4	<0.001	
	Other	536.8 (386.9–745)	10,299 (8137.1–13,035.4)	9762.2	<0.001	
	*p* ^1^	0.468	0.963			
Prior infection	No	568.5 (511.8–631.4)	10,153.4 (9420.7–10,943)	9584.9	<0.001	0.291
	Yes	2118.2 (610.5–7350.1)	20,547.4 (13,580.2–31,089)	18,429.2	<0.001	
	*p* ^1^	0.004	0.003			
Worked in COVID-19 clinic	No	511.7 (453.9–576.8)	9571.4 (8788.5–10,424.2)	9059.7	<0.001	0.002
	Yes	1024.6 (843.1–1245.2)	14,081.8 (12,403.1–15,987.6)	13,057.2	<0.001	
	*p* ^1^	<0.001	<0.001			
Fever	No	510.7 (451.6–577.5)	9192.5 (8423.2–10,032.2)	8681.8	<0.001	0.976
	Yes	878.3 (684.9–1126.3)	15,793.4 (13,567.9–18,384)	14,915.1	<0.001	
	*p* ^1^	<0.001	<0.001			
Any adverse event	No	452.2 (376.3–543.5)	8638.9 (7653.6–9750.9)	8186.7	<0.001	0.346
	Yes	622.2 (541.4–714.9)	10,874.3 (9832.2–12,026.7)	10,252.1	<0.001	
	*p* ^1^	0.007	0.006			

Note. Analysis was conducted with logarithmic transformations. ^1^ *p*-value for group effect; ^2^ *p*-value for time effect; ^3^ effects reported include differences between the groups in the degree of change (repeated-measures ANOVA); ^a.b.c.d^ significant differences among age groups after Bonferroni correction. GMT: Geometric Means Titers, CI: Confidence Interval
